# Association between interleukin-4 gene intron 3 VNTR polymorphism and cancer risk

**DOI:** 10.1186/s12935-014-0131-7

**Published:** 2014-11-30

**Authors:** Yin Duan, Chi Pan, Jinan Shi, Hailong Chen, Suzhan Zhang

**Affiliations:** Cancer Institute (Key Laboratory of Cancer Prevention and Intervention, China National Ministry of Education), The Second Affiliated Hospital, School of Medicine, Zhejiang University, Hangzhou, Zhejiang Province PR China; Department of Medical Oncology, Zhejiang Hospital, Hangzhou, Zhejiang Province PR China

**Keywords:** Cancer, Interleukin-4, Polymorphism, Meta-analysis

## Abstract

**Background:**

Interleukin-4(IL-4) is a critical inflammatory cytokine and has been involved in pathogenesis of cancer. To date, several studies have investigated the association between IL-4 intron 3 variable number of tandem repeats (VNTR) polymorphism and cancer risk in humans; however, the results remain controversial. We performed this meta-analysis to find a more conclusive association between this polymorphism and cancer risk.

**Methods:**

Eight eligible case–control studies were identified through searching electronic databases, including 1583 cases and 1638 controls. Odds ratio (OR) and corresponding 95% confidence interval (CI) were used to estimate the strength of the association.

**Results:**

The results of overall analyses indicated that the variant RP2 allele was associated with a decreased cancer risk compared with the RP1 allele (RP2/RP2 vs. RP1/RP1, OR = 0.64, 95% CI = 0.44-0.94; RP2/RP2 vs. RP1/RP1 + RP1/RP2, OR = 0.75, 95% CI = 0.60-0.92; RP2 vs. RP1, OR = 0.72, 95% CI = 0.56-0.92). In subgroup analyses stratified by ethnicity, there was evidence in the Asian population for an association between this polymorphism and cancer risk (RP2/RP2 vs. RP1/RP1 + RP1/RP2, OR = 0.79, 95% CI = 0.63-0.99; RP2 vs. RP1, OR = 0.77, 95% CI = 0.61-0.97).

**Conclusions:**

IL-4 intron 3 VNTR polymorphism could influence the risk of human cancer. Due to the limitations of this meta-analysis, further well-designed and functional researches should be performed to validate our results.

## Introduction

Cancer is currently a major health burden in the world which results from complicated interactions between genetic and environmental factors [1,2]. Epidemiological studies have revealed that chronic inflammation could pose a risk factor for several cancers [3]. Moreover, inflammation has been linked to the pathogenesis of tumors in up to 15% of human cancers [4]. Cytokines are important inflammatory mediators and there is evidence that human predisposition to cancer could be influenced by polymorphisms located in genes encoding cytokines and their receptors [5].

Interleukin-4(IL-4), produced by activated Th2 type CD4+ T cells, represents a key differentiation cytokine that induces development of Th2 subset of lymphocytes, which is responsible for surveillance and clearance of tumor cells by activation of granulocytes and eosinophils, as well as inhibition of angiogenesis [6,7]. Moreover, Th2 subset is involved in antagonizing IFN-γ function, B cell switching to IgE production, inhibiting macrophage activation and some studies have revealed its anti-tumor activity on several cancers such as colon, breast and renal carcinoma [8,9]. However, IL-4 plays a bilateral role in the control of tumor growth. It has been reported that IL-4 could promote the initiation, progression and spread of head and neck squamous carcinoma [10–13]. Liang et al. [14] have found that there was a significantly higher level of IL-4 mRNA in patients with gastric cancer in stage III and IV than that in stage I and II. In addition, IL-4 also disturbs anti-tumor immunity by down-regulating the expression of Th1 cytokines [10,15] and impairing the CD8+ T cell immune response in the tumor microenvironment [6,11].

The gene encoding IL-4 is located on chromosome 5q31.1 [16]. A rapidly growing number of epidemiologic studies have been conducted to investigate the effect of several IL-4 polymorphisms on human cancer risk. One important polymorphism is located in the intron 3 of IL-4 gene and is composed of a 70-bp sequence of variable number of tandem repeats (VNTR) [17,18]. It has been proved that the IL-4 intron 3 polymorphism might influence the production of IL-4, with the RP1 (two 70-bp repeats) allele enhancing IL-4 expression compared with RP2 (three 70-bp repeats) allele [18,19].

To date, several case–control studies have been carried out to explore the linkage between IL-4 intron 3 VNTR polymorphism and the risk of several human cancers. However, results from different articles remain controversial. We performed this meta-analysis based on the published studies to make a more conclusive evaluation of the association between this polymorphism and cancer risk.

## Materials and methods

### Search strategy

Relevant articles indexed in PubMed and Cochrane Library (from inception to July 30, 2014) were independently searched by two authors, using the key words as follows: (“interleukin-4” or “interleukin 4” or “IL-4” or “IL 4”) and (“tumor” or “cancer” or “carcinoma” or “neoplasm” or “malignancy”) and (“polymorphism” or “polymorphisms” or “SNP” or “variant” or “variation”). The retrieved results were filtered to English language papers. An additional manual search was performed among the references of relevant articles and related articles in PubMed.

### Study identification

All the retrieved articles were reviewed by two authors independently to select studies for inclusion. As for studies with overlapping data reported by the same investigators, the articles with the most complete data were eligible. Studies included in this meta-analysis should meet the following predetermined criteria: 1) case–control design, 2) evaluation of the IL-4 intron 3 VNTR polymorphism and cancer risk, 3) effective estimation of odd ratio (OR) with 95% confidence interval (CI), or enough data to allow calculation of these two statistics. The major criteria for exclusion were: 1) not relevant to IL-4 polymorphism and cancer risk, 2) not with a case–control design (eg, animal studies, reviews, case reports, letters, and editorials), 3) study on inherited cancers, 4) duplicate data.

### Data extraction

Two authors checked each included article independently to extract the necessary data. The following information were collected: first author’s last name, publication year, ethnicity of the study population, country where the study conduct, sample size, source of the control groups (hospital- or population-based), the distribution of variant genotypes of both cases and controls.

### Statistical analysis

Pooled analysis was performed to estimate the strength of the association between IL-4 polymorphism and cancer risk using the odds ratio (OR) with 95% confidence interval (CI). The pooled ORs were calculated by a co-dominant model (RP2/RP2 vs. RP1/RP1, RP1/RP2 vs. RP1/RP1), a dominant model (RP2/RP2 + RP1/RP2 vs. RP1/RP1), a recessive model (RP2/RP2 vs. RP1/RP1 + RP1/RP2), and an allelic model (RP2 vs. RP1). The values of the pooled ORs were tested by Z-test [20]. Hardy-Weinberg equilibrium (HWE) was estimated using goodness-of-fit test based on the chi-square test in the control groups of each study [21].

Heterogeneity among the included studies was evaluated using chi-square based on Q-test and I^2^ statistic [22]. Pooled ORs were calculated using a fixed (Mantel-Haenszel method [23]) or random (DerSimonian-Laird method [24]) effective model according to the absence (I^2^ < 50%) or presence (I^2^ > 50%) of heterogeneity. Sensitivity analyses were conducted by removing one study each time to assess the stability of the results. The potential publication bias of the included studies was evaluated by Begg’s funnel plots graphically and Egger’s linear regression test [25].

All the tests were two sides and it is deemed to be statistically significant when P < 0.05. All the statistical analyses were carried out with Stata/SE software version 12.0 (StataCorp LP, College Station, TX, USA).

## Results

### Literature search and characteristics of eligible studies

**Figure 1:**
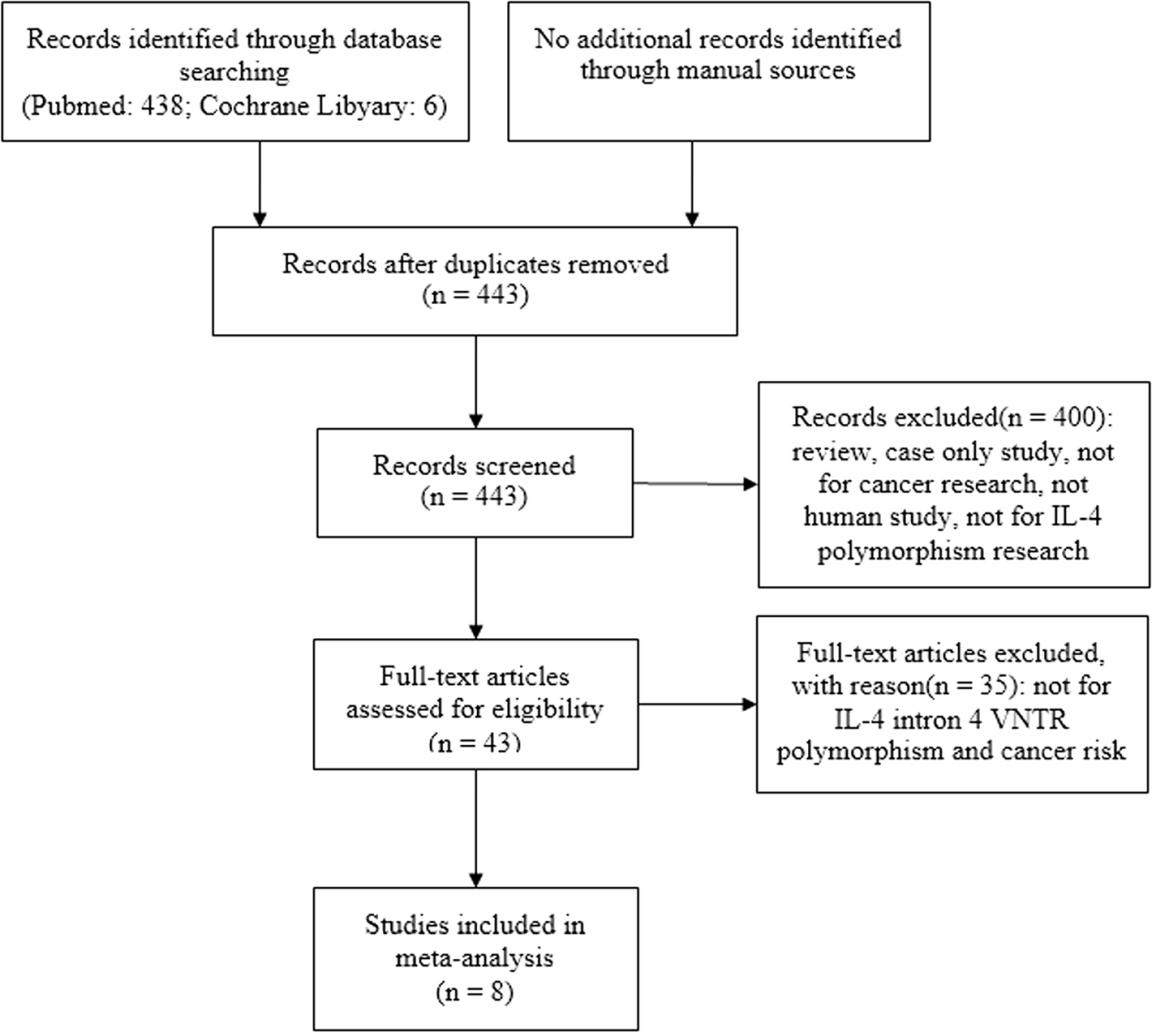
**Flow diagram of the study identification process.**

Our search in electronic databases identified 444 records. Four hundred and one records were excluded after reviewing titles and abstracts and we gained 43 useful studies. After full-text reviewing, we excluded 35 studies and listed the reasons for their exclusion in Figure 1.

There were 8 studies [26–33] suitable for this meta-analysis finally, involving a total of 1583 cases and 1638 controls. Seven of the included studies were conducted on an Asian population and the rest one was on a Caucasian population. Characteristics of eligible studies were summarized in Table 1.

**Table 1:** **Characteristics of eligible studies in the meta-analysis**

**ID**	**First author**	**Year**	**Country**	**Ethnicity**	**Source of controls**	**Cancer type**	**Sample size**		**HWE**
							**Case**	**Control**	
1	Tsai FJ	2005	China	Asian	PB	Bladder cancer	138	105	0.720
2	Tsai MH	2005	China	Asian	HB	Oral cancer	130	105	0.720
3	Lai KC	2005	China	Asian	Not shown	Gastric cancer	123	103	0.961
4	Kesarwani P	2008	India	Asian	HB	Prostate cancer	491	501	0.859
5	Konwar R	2009	India	Asian	HB	Breast cancer	100	200	0.001
6	Shekari M	2011	India	Asian	HB	Cervical cancer	200	200	0.406
7	Yang CM	2014	China	Asian	HB	OPSCC	592	623	0.146
8	Bozdogan T	2014	Turkey	Caucasian	HB	Bladder cancer	100	102	0.199

*PB* population-based controls; *HB* hospital-based controls; *HWE* Hardy-Weinberg equilibrium; *OPSCC* oral and pharyngeal squamous cell carcinoma.

### Quantitative synthesis

**Figure 2:**
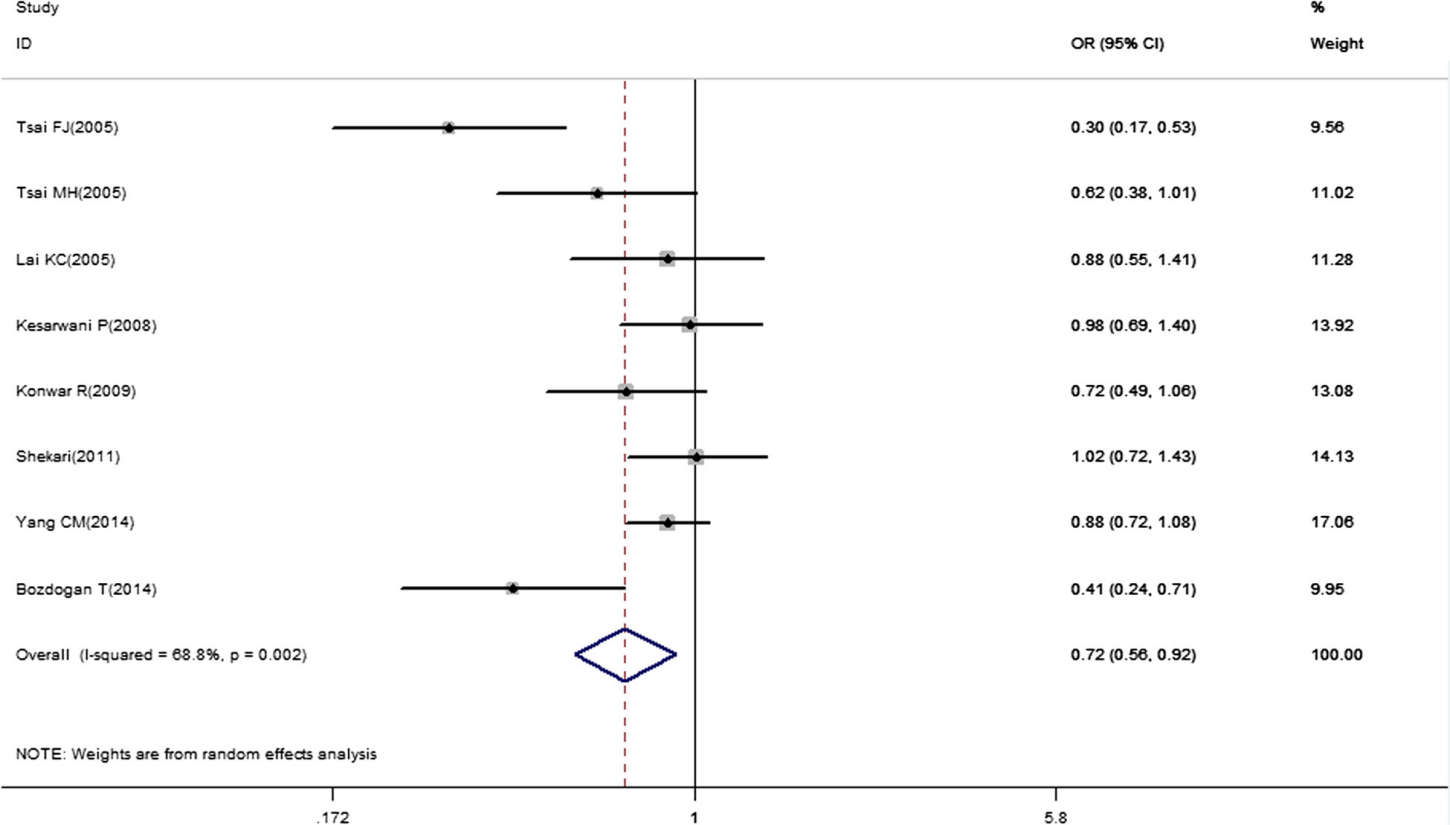
**Forest plot for the overall association between IL-4 intron 3 VNTR polymorphism and cancer risk for an allelic model (RP2 versus RP1).**

The main results of this meta-analysis were presented in Table 2. In the overall analyses, there was an association between the variant genotypes and cancer risk in several genetic models. Significantly decreased cancer risk was observed in the co-dominant model (RP2/RP2 vs. RP1/RP1, OR = 0.64, 95% CI = 0.44-0.94), recessive model (RP2/RP2 vs. RP1/RP1 + RP1/RP2, OR = 0.75, 95% CI = 0.60-0.92), and allelic model (RP2 vs. RP1, OR = 0.72, 95% CI = 0.56-0.92) (Figure 2). In subgroup analyses stratified by ethnicity, there was evidence in the Asian population for an association between this polymorphism and cancer risk (RP2/RP2 vs. RP1/RP1 + RP1/RP2, OR = 0.79, 95% CI = 0.63-0.99; RP2 vs. RP1, OR = 0.77, 95% CI = 0.61-0.97). According to a specific cancer type, declined risk among studies of bladder cancer was found in all genetic models.

**Table 2:** **Pooled analysis of association between IL-4 intron 3 VNTR polymorphism and cancer risk**

**Variables**	**Study**	**N**	**Sample size**		**Test of association**		**Test of heterogeneity**		**Model**	**Egger's test (P-value)**
			**Case**	**Control**	**OR(95% CI)**	**P-value**	**P**_**h**_	**I** ^**2**^ **(%)**		
*RP1/RP2 vs RP1/RP1*	Overall	8	1198	1142	0.79(0.54-1.15)	0.216	0.020	58.0	R	0.631
Ethnicity	Asian	7	1160	1119	0.82(0.57-1.17)	0.264	0.033	56.3	R	0.950
Cancer type	Bladder	2	175	123	**0.26(0.14-0.49)**	0.000	0.297	7.9	F	-
*RP2/RP2 vs RP1/RP1*	Overall	8	1112	1131	**0.64(0.44-0.94)**	0.021	0.158	33.9	F	0.051
Ethnicity	Asian	7	1041	1052	0.75(0.51-1.10)	0.139	0.385	5.5	F	0.225
Cancer type	Bladder	2	191	151	**0.07(0.01-0.38)**	0.002	0.572	0.0	F	-
*RP2/RP2+RP1/RP2 vs RP1/RP1*	Overall	8	1583	1638	0.73(0.49-1.07)	0.102	0.009	62.4	R	0.462
Ethnicity	Asian	7	1483	1536	0.76(0.53-1.08)	0.126	0.022	59.5	R	0.860
Cancer type	Bladder	2	238	207	**0.23(0.13-0.43)**	0.000	0.213	35.5	F	-
*RP2/RP2 vs RP1/RP1+RP1/RP2*	Overall	8	1583	1638	**0.75(0.60-0.92)**	0.007	0.335	12.2	F	0.055
Ethnicity	Asian	7	1483	1536	**0.79(0.63-0.99)**	0.045	0.475	0.0	F	0.043
Cancer type	Bladder	2	238	207	**0.42(0.21-0.85)**	0.044	0.302	6.3	F	-
*RP2 vs RP1*	Overall	8	-	-	**0.72(0.56-0.92)**	0.008	0.002	68.8	R	0.066
Ethnicity	Asian	7	-	-	**0.77(0.61-0.97)**	0.027	0.011	64.0	R	0.159
Cancer type	Bladder	2	-	-	**0.36(0.24-0.53)**	0.000	0.438	0.0	F	-

Note: N number of studies, P_h_ *P*-value of *Q*-test for heterogeneity, R random-effects model, F fixed-effects model, Bold values indicate significant results.

In addition, we omitted 1 study in which the control group was not in agreement with HWE [30] and repeated the meta-analysis. The result was consist with the overall analyses (RP1/RP2 vs. RP1/RP1, OR = 0.74, 95% CI = 0.49-1.13; RP2/RP2 vs. RP1/RP1, OR = 0.62, 95% CI = 0.41-0.95; RP2/RP2 + RP1/RP2 vs. RP1/RP1, OR = 0.71, 95% CI = 0.46-1.09; RP2/RP2 vs. RP1/RP1 + RP1/RP2, OR = 0.78, 95% CI = 0.61-0.98; RP2 vs. RP1, OR = 0.71, 95% CI = 0.53-0.94).

### Test for heterogeneity and sensitivity analysis

**Figure 3:**
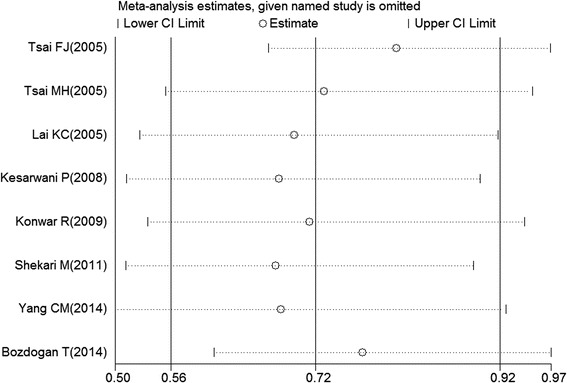
**Sensitivity analysis for the overall association between IL-4 intron 3 VNTR polymorphism and cancer risk for an allelic model (RP2 versus RP1).**

There was significant heterogeneity in the overall analyses for the comparison of the allelic model (RP2 vs. RP1: P = 0.002 and I2 = 68.8% for heterogeneity). Sensitivity analyses were carried out to explore the origin of heterogeneity. Two studies [26,33] were under suspicion and the heterogeneity was remarkably decreased after omitting these two articles (RP2 vs. RP1: OR = 0.87, 95% CI = 0.76-0.99, P = 0.546 and I^2^ = 0.00% for heterogeneity). Moreover, the pooled ORs in all genetic models were not altered qualitatively when any single study was removed, which indicated the results of the present meta-analysis were relatively stable and credible (Figure 3).

### Publication bias

**Figure 4:**
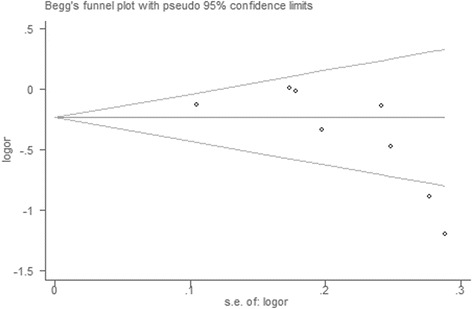
**Begg’s funnel plot for the overall association between IL-4 intron 3 VNTR polymorphism and cancer risk for an allelic model (RP2 versus RP1).**

The publication bias of eligible literatures was assessed by Begg’s funnel plots graphically and Egger’s test statistically. The shapes of funnel plots in all models did not show any evidence of an obvious asymmetry (Figure 4). Meanwhile, the Egger’s test indicated no publication bias either (RP1/RP2 vs. RP1/RP1: P = 0.631, RP2/RP2 vs. RP1/RP1: P = 0.051, RP2/RP2+ RP1/RP2 vs. RP1/RP1: P = 0.462, RP2/RP2 vs. RP1/RP1 + RP1/RP2: P = 0.055, RP2 vs. RP1: P = 0.066).

## Discussion

Cancer is a heterogeneous disease resulting from genetics, environmental factors, and their interactions, the clinical outcome of which is difficult to predict [34–36]. Inflammation has been associated with variant malignancies. Elevated levels of inflammatory cytokines have been linked to inflammatory exacerbation. As a critical anti-inflammatory cytokine, IL-4 has implicated in the pathogenesis of various cancers. Elevated plasma levels of IL-4 were correlated with risk of acute myeloid leukemia, melanoma, head and neck squamous cell carcinoma, non-small-cell lung cancer, prostate, colon, breast, renal cell cancer [37–40], and were associated with poor prognosis in non-Hodgkin lymphoma [41]. More specifically, several polymorphisms of the gene encoding IL-4 have been proved to be risk factors for cancer [33,42–45]. Besides the most common polymorphisms, −590C > T(rs2243250) and -33 T > C(rs2070874), the intron 3 VNTR polymorphism also plays an important role in alteration of IL-4 production.

To our knowledge, Li et al. performed a meta-analysis about the association of IL-4 intron 3 VNTR polymorphism and cancer risk [46]. Their results showed that there was no association between this polymorphism and cancer risk. However, some concerns were related to this meta-analysis. First, the sample size was relatively small, involving 791 cases and 772 controls from 5 case–control studies. Second, there was significant heterogeneity and the origin of heterogeneity was not well explored. In addition, several important studies about the association of IL-4 intron 3 VNTR polymorphism and cancer risk have been published recently.

The present meta-analysis, including 1583 cases and 1638 controls from 8 case–control studies, comprehensively assessed the association between IL-4 intron 3 VNTR polymorphism and risk of human cancers. We found an association between IL-4 intron 3 VNTR polymorphism and cancer risk. Our results indicated the RP1 allele might be a risk factor for malignancies. The results were robust because the pooled ORs did not alter in sensitivity analyses. Stratified analyses suggested that the association was mainly in Asian populations and in bladder cancer. Since there was significant heterogeneity in several genetic models, two studies were founded out by sensitivity analyses. We re-checked these 2 studies and found that 1 study [26] was the only population-based design in the 8 included studies and the other one [33] was the only one that conducted on a Caucasian population. In addition, we pooled the data from studies on diverse cancer types, which might contribute to some extent of the heterogeneity. Different cancer types might give rise to different host responses, and the interactions between different environmental factors and host might also influence the susceptibility to different cancer types. Because of the limited study number, we could not perform a meta-regression analysis to estimate the extent of heterogeneity from different cancer types.

Some limitations of our meta-analysis should be pointed out. First, the pooled outcomes were calculated based on unadjusted estimates, which limited a more precise evaluation on adjusted estimates by several important factors like age, sex, lifestyle and etc. Only one study reported that IL-4 intron 3 VNTR polymorphism was associated with oral and pharyngeal carcinoma risk, which interacted with alcohol drinking [32]. Thus, lacking of these original information limited supplementary assessment of the potential interactions because gene-gene, gene-environment interactions and other polymorphisms of the same gene might influence cancer predisposition. Second, most of the studies included in the present meta-analysis only focused on the relationship between IL-4 intron 3 VNTR polymorphism and cancer risk, which made it hard to assess the effects of IL-4 haplotypes composed of different IL-4 polymorphisms on carcinogenesis. There was evidence that IL-4 -590C > T(rs2243250), −33 T > C(rs2070874) polymorphisms were associated with cancer risk [42,43,45]. Thus, the status of other IL-4 polymorphisms might cover up the impact of intron 3 VNTR polymorphism on carcinogenesis. Third, limited study number restricted us to perform additional subgroup analyses. Forth, although there was no publication bias statistically, the potential publication bias might exist because some studies with negative results were not published. Moreover, there was only one study conducted on the Caucasian population and no study on the African population in this meta-analysis.

Despite these limitations, advantages in our meta-analysis should be also acknowledged. First, the statistical power was definitely increased by pooling a substantial number of cases and controls. Second, all the eligible studies met the inclusion and exclusion criteria strictly and completely. Third, no publication bias was detected through Begg’s funnel plots and Egger’s test, which indicated that the pooled results should be unbiased.

In conclusion, the results of this meta-analysis were statistically credible. The relationship between IL-4 intron 3 VNTR polymorphism and cancer risk was assessed and this polymorphism was associated with cancer risk, mainly in Asians. To draw a more precise conclusion, further studies should be carried out with more detailed individual information, concerning the effects of other polymorphisms and haplotypes, enrolling larger sample size of cases and well-matched controls, especially in Caucasians and Africans, to validate the role of IL-4 polymorphism in carcinogenesis.
